# Grafting C8-C16 alkyl groups altered the self-assembly and curcumin –loading properties of sodium caseinate in water

**DOI:** 10.1016/j.dib.2017.11.039

**Published:** 2017-11-13

**Authors:** Yaqiong Zhang, Puyu Yang, Fangyi Yao, Jie Liu, Liangli (Lucy) Yu

**Affiliations:** a Institute of Food and Nutraceutical Science, School of Agriculture and Biology, Shanghai Jiao Tong University, Shanghai 200240, China; b Beijing Advanced Innovation Center for Food Nutrition and Human Health, Beijing Technology & Business University (BTBU), Beijing 100048, China; c Department of Nutrition and Food Science, University of Maryland, College Park, MD 20742, United States

**Keywords:** Caseinate, Alkylated caseinate, Self-assembly, Curcumin-loading property

## Abstract

The data presented here are related to the research article entitled “Synthesis and characterization of alkylated caseinate, and its structure-curcumin loading property relationship in water” (Zhang et al., 2018) [1]. This data article reports the detailed spectra information for ^1^H NMR, ^13^C NMR and UPLC-Q-TOF MS of the N-succinimidyl fatty acid esters with various alkyl chain lengths (Cn-NHSs, *n* = 8, 12, 14 and 16). ^1^H NMR, ^13^C NMR and UPLC-Q-TOF MS spectra for C16-NHS are shown as an example. Then the stacked ^1^H NMR spectra of the obtained alkylated caseinates (Cn-caseinates, *n* = 8, 12, 14 and 16) are provided. The surface hydrophobicity index (S_0_) of Cn-caseinates with different substitution degrees (SD) of alkyl groups is shown. Additionally, Visual appearances for the formed aqueous dispersions of curcumin-loaded native caseinate (NaCas) and Cn-caseinates self-assemblies are shown. X-ray diffraction patterns of curcumin, C16-caseinate, its physical mixture and curcumin-loaded C16-caseinate self-assemblies are examined. The re-dispersibility and short-term storage stability of the curcumin-loaded NaCas and C16-caseinate self-assemblies are also studied.

**Specifications Table**

**Table:** 

Subject area	*Chemistry*
More specific subject area	*Effect of alkylation on the self-assembly and curcumin-loading properties of sodium caseinate in water*
Type of data	*Figures and text files*
How data was acquired	*The structural confirmations of Cn-NHSs and Cn-caseinates were determined by*^*1*^*H NMR (AVANCE III 600 MHz, Bruker, Karlsruhe, Germany),*^*13*^*C NMR (AVANCE III 600 MHz, Bruker, Karlsruhe, Germany) and UPLC-Q-TOF MS (Xevo G2,Q-TOF mass spectrometer, Waters, Milford, MA, USA);*
	*The surface hydrophobicity index (S*_*0*_*) of Cn-caseinates was measured by multilabel plate reader (M200 PRO, Tecan Group Ltd., Mannedorf, Switzerland);*
	*The existing state of curcumin was analyzed by X-ray diffraction (RD-D8 ADVANCE DA VINCI, Bruker, Karlsruhe, Germany);*
	*The short-term storage stability of curcumin-loaded caseinate self-assemblies was measured by dynamic light scattering (DLS) method (Zetasizer Nano ZS90, Malvern Instruments Ltd., Worcestershire, UK).*
Data format	*Raw and analyzed*
Experimental factors	*Sodium caseinate and alkylated caseinates containing selected C8-C16 alkyl groups*
Experimental features	*The effect of alkylation on the self-assembly and curcumin-loading properties of sodium caseinate was determined.*
Data source location	*Shanghai, China*
Data accessibility	*The data are available within this article.*

**Value of the data**

1) The detailed structural characterization data of alkylated caseinates could provide a reference to other researchers in identifying modified proteins.
2) The ^1^H-NMR method is valuable for quantifying the substitution degrees of alkyl groups grafted in water-soluble proteins.
3) Curcumin-loaded Cn-caseinate self-assemblies were prepared using the dialysis method and this method could be compared to other preparation methods for protein self-assemblies.

## 1 Data

**Fig. 1:**
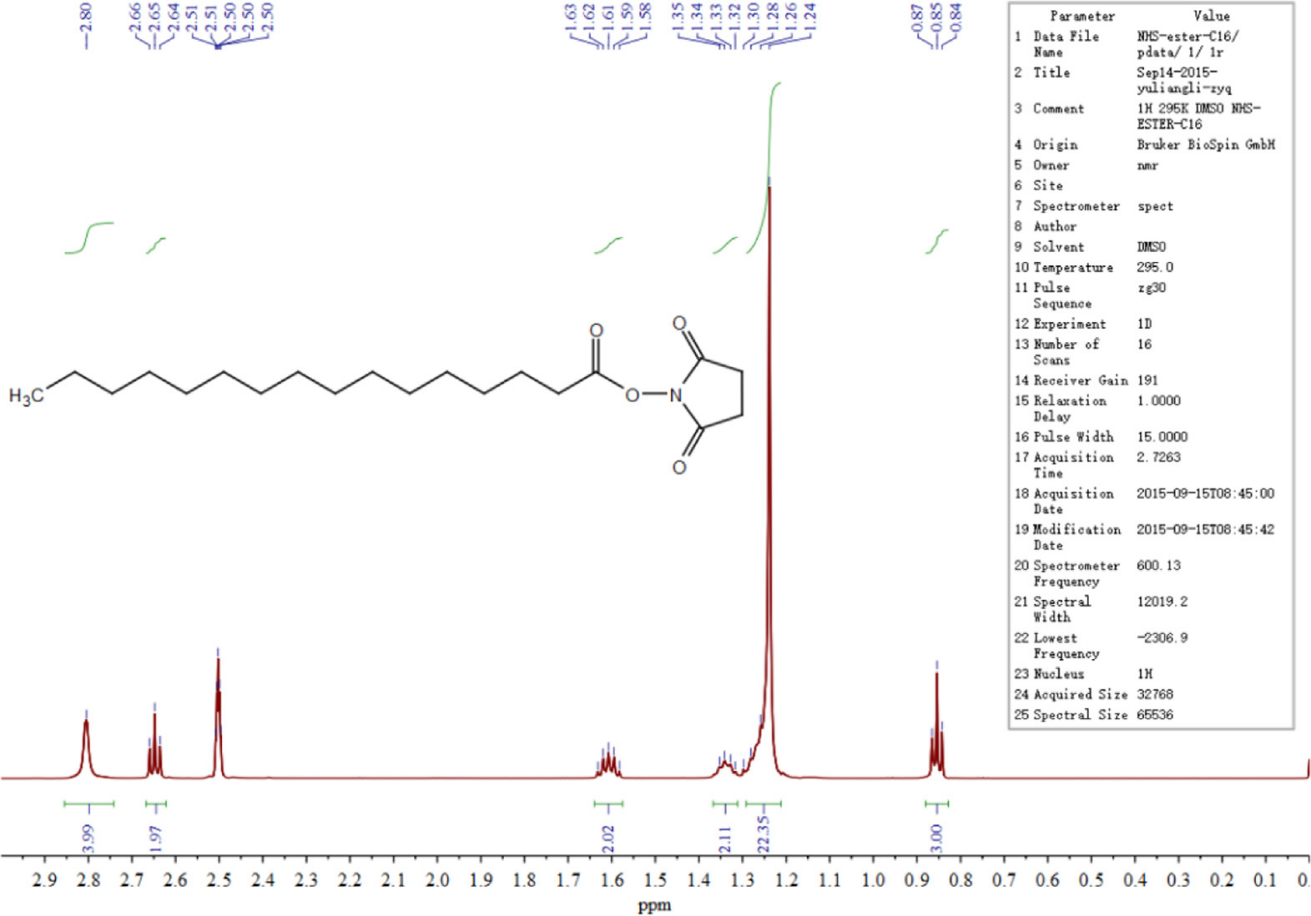
^1^H NMR spectrum of C16-NHS.

**Fig. 2:**
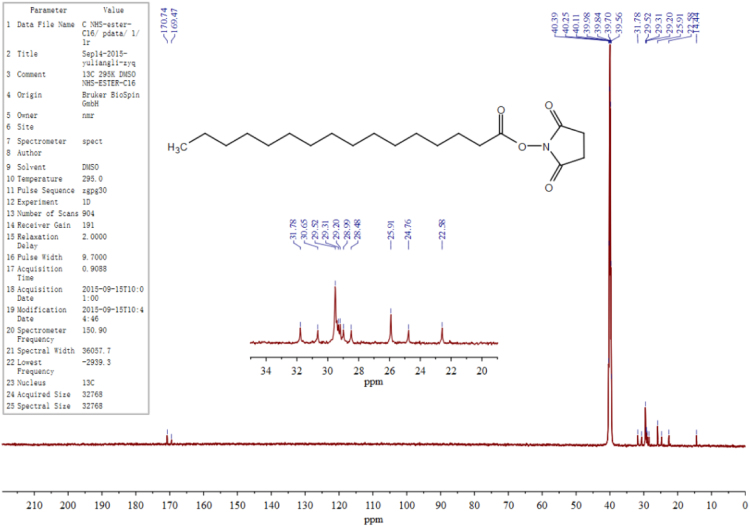
^13^C NMR spectrum of C16-NHS.

**Fig. 3:**
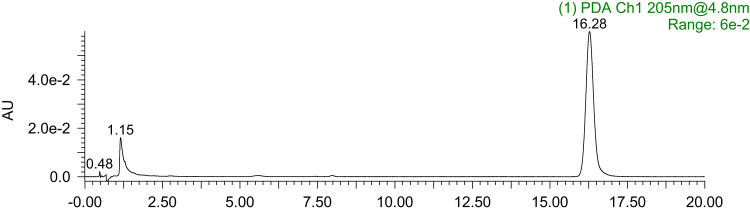
UPLC spectrum of C16-NHS.

**Fig. 4:**
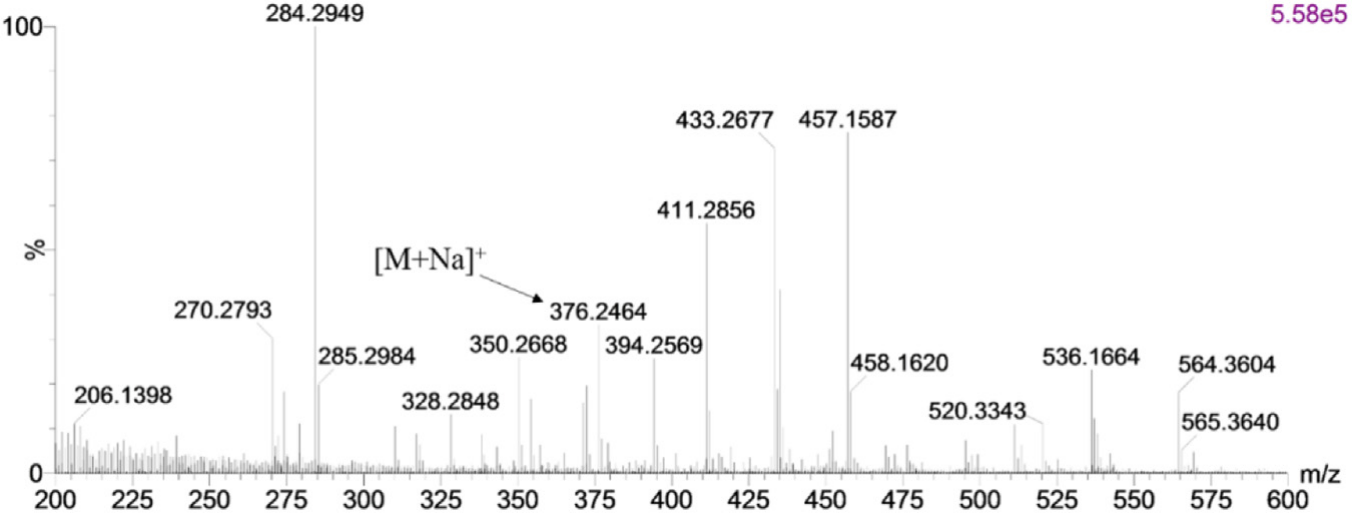
MS spectrum of C16-NHS.

The data presented in Section 1.1 include ^1^H NMR, ^13^C NMR and UPLC-Q-TOF MS spectra information of the obtained *N*-succinimidyl fatty acid esters with various alkyl chain lengths (Cn-NHSs: C8-NHS, C12-NHS, C14-NHS, C16-NHS). As an example, ^1^H NMR, ^13^C NMR and UPLC-Q-TOF MS spectra for C16-NHS are shown in Fig. 1, Fig. 2, Fig. 3, Fig. 4**,** respectively.

**Fig. 5:**
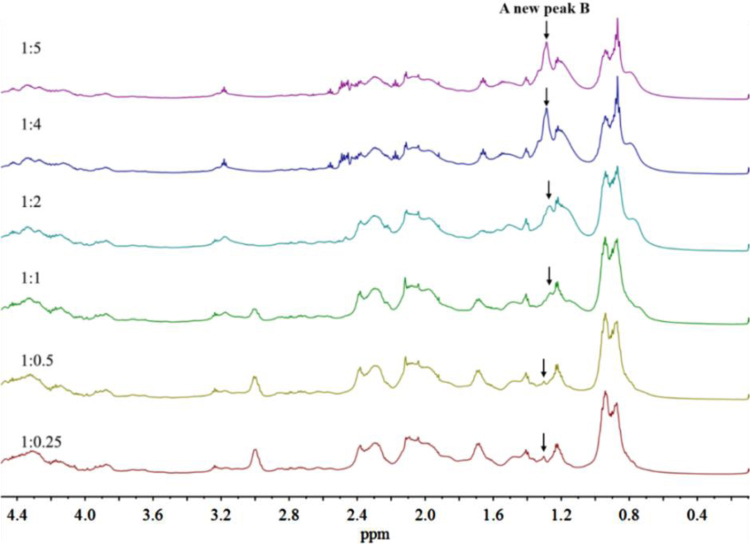
Stacked ^1^H NMR spectra of C8-caseinate.

**Fig. 6:**
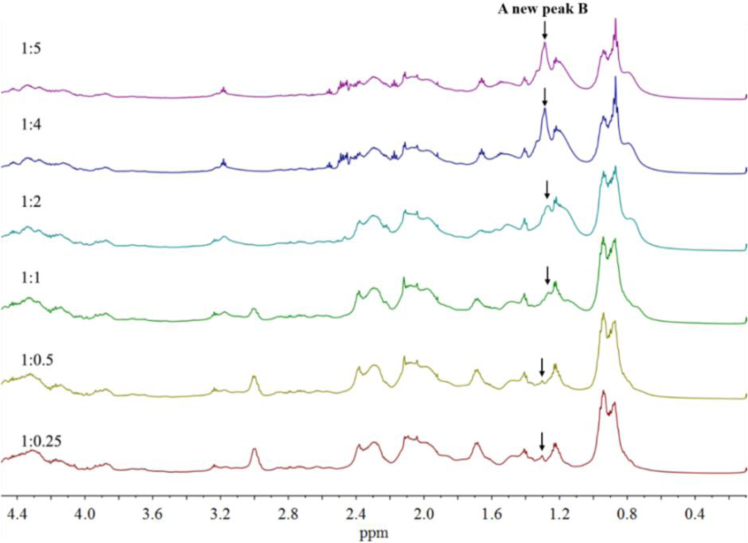
Stacked ^1^H NMR spectra of C12-caseinate.

**Fig. 7:**
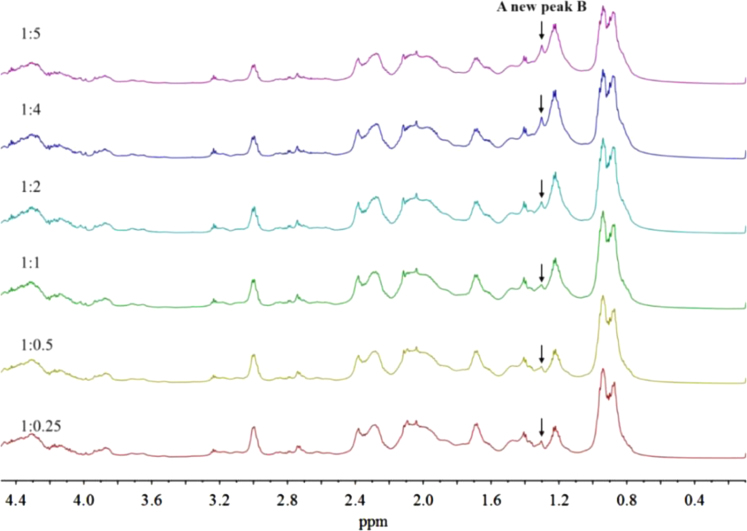
Stacked ^1^H NMR spectra of C14-caseinate.

**Fig. 8:**
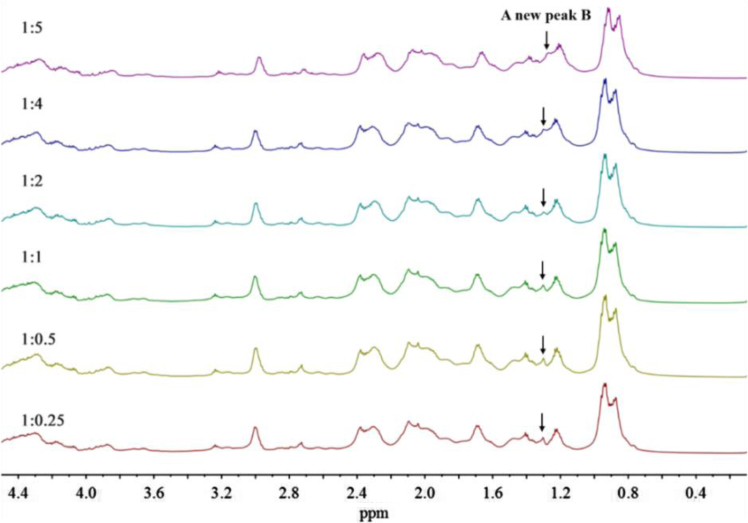
Stacked ^1^H NMR spectra of C16-caseinate.

In Section 1.2 are presented data on the stacked ^1^H NMR spectra of the alkylated caseinates (Cn-caseinates): C8-caseinate (Fig. 5), C12-caseinate (Fig. 6), C14-caseinate (Fig. 7) and C16-caseinate (Fig. 8). Cn-caseinates with different substitution degrees (SD) of alkyl groups were obtained under different feeding molar ratios (1:0.25, 1:0.5, 1:1, 1:2, 1:4 and 1:5) of the ε-NH_2_ in lysyl residue of native caseinate (NaCas) to Cn-NHS.

**Fig. 9:**
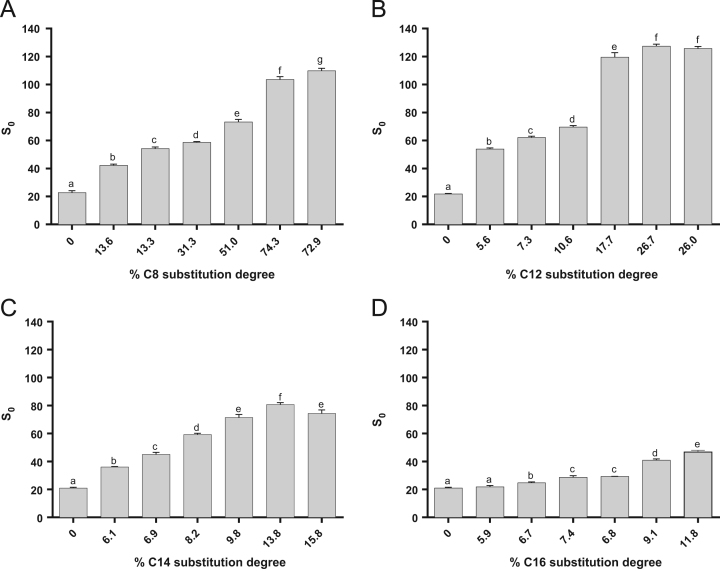
Surface hydrophobicity index (*S*_0_) of A) C8-caseinates, B) C12-caseinates, C) C14-caseinates, and D) C16-caseinates with different substitution degrees of alkyl groups. Different letters (a-g) above the columns indicate that the data are significantly different (*P* < 0.05).

**Fig. 10:**
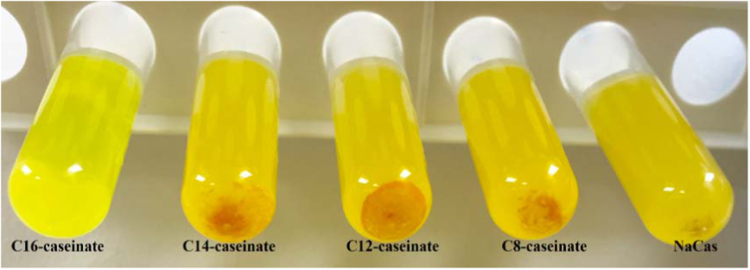
Visual appearances for the formed aqueous dispersions of curcumin-loaded NaCas and Cn-caseinates self-assemblies.

**Fig. 11:**
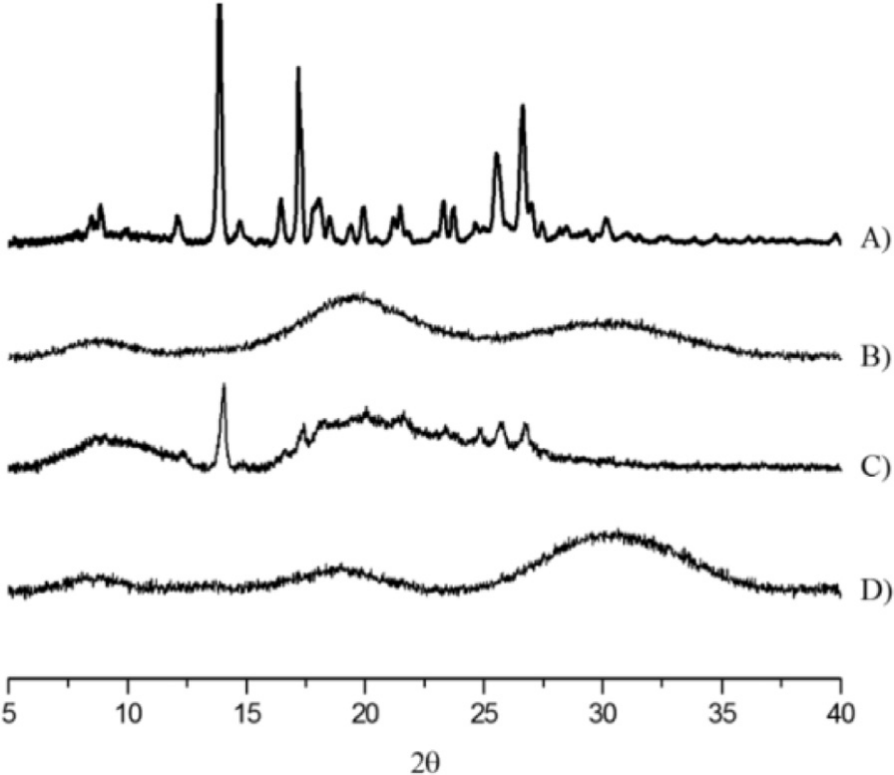
X-ray diffraction patterns of curcumin (A), C16-caseinate (SD of 7.4%) (B), its physical mixture (C) and curcumin-loaded C16-caseinate self-assemblies (D).

**Fig. 12:**
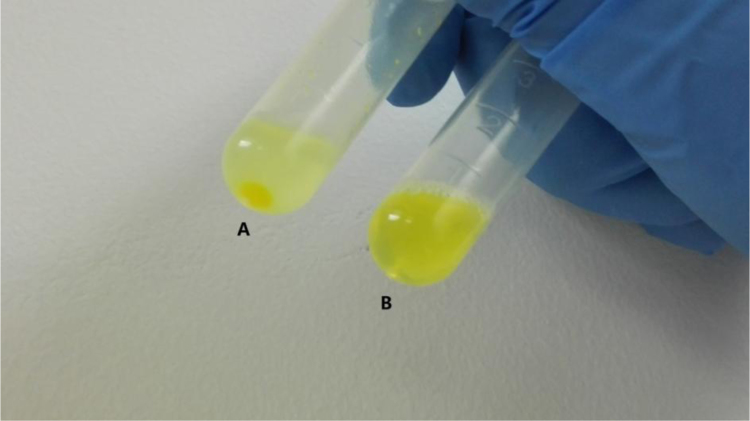
Re-dispersibility of curcumin-loaded NaCas (A) and C16-caseinate (B) self-assemblies.

**Fig. 13:**
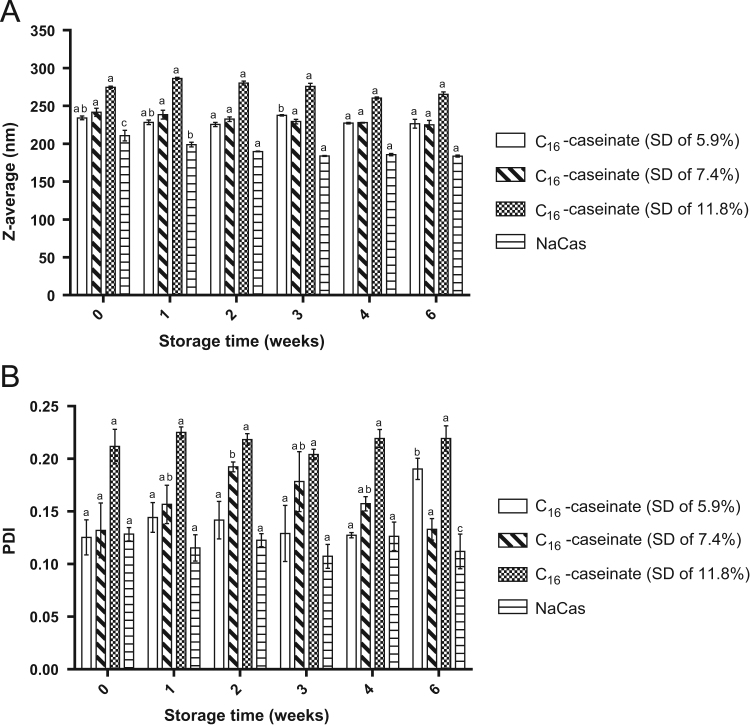
The size (A) and size distribution (B) of curcumin-loaded NaCas and C16-caseinate self-assemblies.

The data presented in Section 1.3 include the surface hydrophobicity, self-assembly and curcumin-loading properties of NaCas and Cn-caseinates in water. Fig. 9 shows the surface hydrophobicity index (*S*_0_) of Cn-caseinates with different substitution degrees (SD) of alkyl groups. Visual appearances for the formed aqueous dispersions of curcumin-loaded NaCas and Cn-caseinates is shown in Fig. 10. Fig. 11 provides the X-ray diffraction patterns of curcumin, C16-caseinate, its physical mixture and curcumin-loaded C16-caseinate self-assemblies. The re-dispersibility and short-term storage stability of curcumin-loaded NaCas and C16-caseinate self-assemblies in water are also shown in Fig. 12, Fig. 13, respectively.

### 1.1 ^1^H NMR, ^13^C NMR and UPLC-Q-TOF MS spectra information for Cn-NHSs (*n* = 8, 12, 14 and 16)

C8-NHS: ^1^H NMR (600 MHz, d_6_-DMSO) *δ* 2.81 (s, 4H), 2.65 (t, J = 6.9 Hz, 2H), 1.61 (p, J = 6.9 Hz, 2H), 1.34 (dd, J = 6.7 Hz, 2H), 1.31–1.22 (m, 6H), 0.86 (t, J = 6.3 Hz, 3H). ^13^C NMR (150 MHz, d_6_-DMSO) *δ* 170.73 (2 × C=O), 169.46, 31.53, 30.65, 28.64, 28.43, 25.90 (2 × CH2), 24.76, 22.46, 14.39. UPLC: *t*_R_ = 1.49 min; HRMS (ESI, positive mode): [M + H]^+^ *m*/*z* 242.1392 (Calcd for C_12_H_20_O_4_N, 242.1392), [M + Na]^+^ *m*/*z* 264.1212 (Calcd for C_12_H_20_O_4_N, 264.1212).

C12-NHS: ^1^H NMR (600 MHz, d_6_-DMSO) *δ* 2.81 (s, 4H), 2.65 (t, J = 7.3 Hz, 2H), 1.64 – 1.58 (m, 2H), 1.34 (dd, J = 6.9 Hz, 2H), 1.30 – 1.22 (m, 14H), 0.86 (t, J = 7.0 Hz, 3H). ^13^C NMR (150 MHz, d_6_-DMSO) *δ* 170.23 (2 × C=O), 168.96, 31.30, 30.17, 28.99, 28.95, 28.84, 28.72, 28.52, 28.00, 25.42 (2 × CH2), 24.28, 22.11, 13.94. UPLC: *t*_R_ = 4.07 min; HRMS (ESI, positive mode): [M + Na]^+^ *m*/*z* 320.1835 (Calcd for C_16_H_27_O_4_NNa, 320.1838).

C14-NHS: ^1^H NMR (600 MHz, d_6_-DMSO) *δ* 22.81 (s, 4H), 2.65 (t, J = 7.2 Hz, 2H), 1.64 – 1.58 (m, 2H), 1.34 (dd, J = 6.9 Hz, 2H), 1.29 – 1.22 (m, 18H), 0.85 (t, J = 7.0 Hz, 3H). ^13^C NMR (150 MHz, d_6_-DMSO) *δ* 170.71 (2 × C=O), 169.44, 31.79, 30.65, 29.55, 29.51 (2 × CH2), 29.43, 29.32, 29.21, 29.00, 28.49, 25.90 (2 × CH2), 24.76, 22.59, 14.42. UPLC: *t*_R_ = 7.96 min; HRMS (ESI, positive mode): [M + Na]^+^ *m*/*z* 348.2148 (Calcd for C_18_H_31_O_4_NNa, 376.2464).

C16-NHS: ^1^H NMR (600 MHz, d_6_-DMSO) *δ* 2.80 (s, 4H), 2.65 (t, J = 7.2 Hz, 2H), 1.64 – 1.57 (m, 2H), 1.33 (dd, J = 7.0 Hz, 2H), 1.29 – 1.21 (m, 22H), 0.85 (t, J = 7.0 Hz, 3H). ^13^C NMR (150 MHz, d_6_-DMSO) *δ* 170.74 (2 × C=O), 169.47, 31.78, 30.65, 30.01 – 29.10 (8 × CH2), 28.99, 28.48, 25.91 (2 × CH2), 24.76, 22.58, 14.44. UPLC: *t*_R_ = 16.28 min; HRMS (ESI, positive mode): [M + Na]^+^ *m*/*z* 376.2464 (Calcd for C_20_H_35_O_4_NNa, 404.2777).

Shown as an example, see Fig. 1, Fig. 2, Fig. 3, Fig. 4 for the ^1^H NMR, ^13^C NMR and UPLC-Q-TOF MS spectra for C16-NHS.

### 1.2 The stacked ^1^H NMR spectra of Cn-caseinates (*n* = 8, 12, 14 and 16)

See Fig. 5, Fig. 6, Fig. 7, Fig. 8.

### 1.3 Surface hydrophobicity, self-assembly and curcumin-loading properties of NaCas and Cn-caseinates in water

See Fig. 9, Fig. 10, Fig. 11, Fig. 12, Fig. 13.

## 2 Experimental design, materials and methods

The methodologies that allowed the data presented here are described in the cited reference [1].
